# Rapid drug resistance prediction in positive *Mycobacterium tuberculosis* clinical samples using an extensive targeted next-generation sequencing panel

**DOI:** 10.1080/22221751.2026.2627072

**Published:** 2026-02-03

**Authors:** Ebba Rosendal, Joana Isidro, Sofia Carneiro, João Paulo Gomes, Rita Macedo

**Affiliations:** a ECDC Fellowship Programme, Public Health Microbiology path (EUPHEM), European Centre for Disease Prevention and Control (ECDC), Stockholm, Sweden; b Infectious Diseases Department, National Institute of Health Doctor Ricardo Jorge, Lisbon, Portugal; c Genomics and Bioinformatics Unit, Infectious Diseases Department, National Institute of Health Doctor Ricardo Jorge, Lisbon, Portugal; d National Reference Laboratory for Mycobacteria, Infectious Diseases Department, National Institute of Health Doctor Ricardo Jorge, Lisbon, Portugal; e Animal and Veterinary Research Center (CECAV), Faculty of Veterinary Medicine, Lusófona University – Lisbon University Centre, Lisbon, Portugal

**Keywords:** *Mycobacterium tuberculosis*, TB, antimicrobial resistance, targeted sequencing, drug susceptibility testing, Nanopore sequencing

## Abstract

Tuberculosis (TB) remains a global health challenge, exacerbated by the emergence of drug-resistant *Mycobacterium tuberculosis* strains. Most methods for drug susceptibility testing (DST) are culture-dependent and time consuming, possibly delaying optimal TB-treatment. This study aimed to develop an extensive targeted next-generation sequencing (tNGS) approach for rapid genotypic DST directly from clinical samples. We designed a tNGS panel comprising 30 amplicons targeting 19 genomic regions associated with resistance to 20 antibiotics. This method was applied to 71 smear-positive (0–3+) pulmonary TB clinical samples collected at the Portuguese National Reference Laboratory. DNA was extracted and amplified using multiplex PCRs, followed by sequencing on Oxford Nanopore Technologies MinION platform. Sequencing data were using TB-Profiler and the tNGS results compared to phenotypic DST and whole genome sequencing (WGS) data from corresponding isolates. The tNGS demonstrated high concordance with both phenotypic and WGS-based DST across different sample types and smear positivity levels. For first-line drugs, tNGS showed 88% categorical agreement (CA) with pDST, increasing to 97% when excluding undetermined results. Compared to WGS across all analysed antibiotics, tNGS achieved 92% CA, increasing to >99% when excluding undetermined results. Validation of the tNGS panel showed 90% (1,895/2,076) of amplicons reaching >10x coverage at all analysed positions and 43 (61%) samples with all complete amplicons above this threshold. Non-specific amplification of contaminant bacterial DNA was minimal, with most mapped off-target reads being of human origin. This method enables comprehensive resistance prediction directly from clinical samples and signifies an important development in TB diagnostics and resistance monitoring.

## Introduction

Tuberculosis (TB) remains one of our most urgent public health concerns. Active TB disease primarily manifests as pulmonary disease with high mortality if left untreated, resulting in approximately 1.25 million deaths in 2023 [1]. TB-treatment is complicated by the emergence of drug-resistant *Mycobacterium tuberculosis* strains [1,2]*.* Rapid diagnosis, including drug susceptibility testing (DST), is crucial for informing optimal treatment regimens and preventing further resistance development.

The gold standard for *M. tuberculosis* DST is culture-based phenotypic testing (pDST). While reliable and well-established, it is limited by long turn-around time (2–3 months) and laboratory biosafety requirements (BSL-3), potentially delaying optimal TB treatment. While whole genome sequencing (WGS) for *M. tuberculosis* may provide identification and genotypic DST (gDST) of early positive cultures [3,4], it is still limited by the need for culture. Attempts to enrich for *M. tuberculosis* genomes directly from clinical samples, prior to sequencing (e.g. molecular capture), have proven useful but remain limited by sensitivity issues and relatively high costs [5,6].

To overcome some of these limitations, targeted molecular diagnosis solutions for use directly on clinical samples have been developed, including real-time PCR assays, line probe assays (LPAs), hybridization-based assays and targeted next-generation sequencing (tNGS) [7,8]. These assays typically focus on well-characterized resistance-associated targets, such as *rpoB* (rifampicin), *katG* and *inhA* (isoniazid), *gyrA/gyrB* (fluoroquinolones), *pncA* (pyrazinamide) and *rrs* (aminoglycosides) [9]. As sequencing technology becomes more advanced and cost-effective, tNGS is emerging as a scalable and sensitive option for gDST of *M. tuberculosis* [10,11], offering broader mutation detection and improved hetero-resistance identification compared to other molecular assays. The World Health Organization (WHO)'s 2024 consolidated guidelines for TB endorses two commercial tNGS-based kits (Deeplex® Myc-TB and AmPORE-TB®) analysing resistance against six to 10 selected first- and second-line antibiotics [12].

While reducing the time to results, current tNGS solutions often target a limited number of short genomic regions (so-called hot-spots) focusing on a restricted number of drugs [12]. Additionally, the available commercial kits may be too costly for widespread use in low- or medium-resource settings, and do not allow for context-specific adjustment of targets. Also, the commercial kits often provide no information on the specific mutations detected and sometimes have limited ability to detect subpopulations or hetero-resistance. Together, this highlights the need for development of alternative, low-cost tNGS solutions that enable broader and more accessible DST for *M. tuberculosis*.

In this study, we developed a tNGS approach for gDST of *M. tuberculosis* including 30 amplicons, targeting 19 genomic regions of interest with known resistance-associated mutations for 20 antibiotics used for the treatment of TB. We evaluated this panel on 71 clinical pulmonary TB samples with varying resistance profiles, using both pDST and WGS data as reference.

## Methods

### Clinical samples

We used smear-positive samples from patients diagnosed with pulmonary TB in Portugal between 2020 and 2024. Samples were selected based on availability, to maximize the representation of sample types and phenotypic resistance patterns. These included sputum samples (n = 54), bronchial secretions (n = 10), bronchial lavage (n = 3), respiratory secretions (n = 1), respiratory secretions aspirate (n = 1), bronchoalveolar lavage (n = 1) and tracheal aspirate (n = 1). These were collected as part of routine diagnostics at the National Reference Tuberculosis Laboratory (NRL-TB) of the Portuguese National Institute of Health (NIH). Samples were decontaminated using N-acetyl-cisteine-NaOH BD BBL^™^ MycoPrep^™^ kit according to manufacturer´s instructions (Becton Dickinson Company, East Rutherford, NJ, USA) and used as inoculum for liquid and solid cultures and subsequent pDST. Samples were analysed by microscopy and visually quantified according to WHO guidelines to acid fast bacilli (AFB) scoring (1+, 2+, and 3+) with the addition of 0 + defined as 1–9 AFB/100 microscopic fields. The remaining samples were kept at −20°C until DNA extraction.

### pDST

All isolates were subjected to pDST against the first-line drugs rifampicin (RIF), isoniazid (INH), ethambutol (EMB), pyrazinamide (PZA), and streptomycin (STM). Isolates that were found to be resistant to at least RIF and INH are considered multidrug-resistant TB (MDR-TB) and were subjected to pDST against kanamycin (KAN), amikacin (AMK), capreomycin (CAP), ethionamide (ETH), para-aminosalicylate sodium (PAS), cycloserine (CYCLO), linezolid (LZD), bedaquiline (BDQ), clofazimine (CFZ), delamanid (DLM), moxifloxacin (MFX), levofloxacin (LFX), ofloxacin (OFX), ciprofloxacin (CIP). pDST was carried out on an automated liquid medium-based system, Bactec MGIT960 (Becton Dickinson, East Rutherford, NJ, USA), using the following drug concentrations (µg/ml): for STR:1.0; for INH:0.1; for RIF:1.0/0.5 (critical concentration was updated to 0.5 µg/ml according to the WHO revision in February 2021); for EMB:5.0; for PZA:100.0; for OFX:2.0; for AMK:1.0; for CAP:2.5; for KAN:5.0; for ETH:5.0; for PAS:4.0; for CYCLO:16.0; for LZD:1.0; for BDQ:1.0; for CFZ:1.0; for DLM:0.06; for MFX:0.25; for LFX:1.0.

### DNA extraction

Decontaminated samples were subjected to heat inactivation at 95°C for 30 min. DNA was extracted using the QIAamp DNA Mini Kit (Qiagen, Düsseldorf, Germany) in accordance with the protocol for total DNA extraction from tissues; 250 µl of clinical sample in lysis buffer (ATL) and proteinase K was incubated rocking overnight at 56°C before extraction following the manufacturer’s instructions. DNA concentrations were determined using the Qubit 4 Fluorometer (Thermo Fisher Scientific, Waltham, MA, USA) with the Qubit dsDNA High Sensitivity (HS) Assay Kit.

### Primer design and multiplex PCRs

Primers were designed using Primer3 [13] via Benchling (https://www.benchling.com/), targeting regions at least 100 nucleotides away from known resistance-conferring mutations. Amplicons were aimed at 900–1100 bp and primers at 22 bp with a melting temperature of approx. 64°C. To prevent off-target amplification, primer candidates were manually checked for cross-reactivity against the human genome using the UCSC Genome Browser [14] and screened using BLAST + [15].

Multiplex PCR pools aimed for 10–12 primers per reaction, to minimize primer-dimers (predicted using Thermo Fisher Scientific online tool, https://www.thermofisher.com) and optimize annealing temperature compatibility and amplification efficiency as determined by optimization of individual PCRs (using DNA from pure cultures). Two primer pairs were redesigned from initial sequences: *embB* and *rrs* to improve amplification efficiency (*embB*) or specificity for the *M. tuberculosis* complex over other bacterial species (*rrs*). Annealing temperatures were optimized for weakly amplifying or non-specific primer pairs and primer concentrations in the multiplex pools were adjusted to achieve balanced sequencing coverage across targets. From first design to final protocol, 4–5 rounds of individual PCR optimization and 3–4 rounds of multiplex pool optimization were performed. Alltogether, optimization resulted in three multiplex PCRs (primer details in Table S1). Notably, the protocol was updated during optimization and only 35 of the 71 samples were amplified using the final optimized protocol containing *embB*.

PCRs were run with HotStarTaq Plus Master Mix (Qiagen, Germany), reaction volume 50 µl, using 10 µl 5x Q-solution (Quiagen, Germany) and 12.6 µl DNA per reaction. The PCRs were screened for bands on an agarose gel (1% agarose, 100 V, 40 min) prior to library preparation. Bands were visually classified as strong, medium, weak, or very weak/no band, and these classifications determined the mixing ratios for the three pools, specifically, the medium, weak and very weak bands would be pooled at 1.5X, 3X and 6X, respectively, relative to the strong band (1X) volume.

### Library preparation

The Native Barcoding Kit 24 v14 (SQK-NBD114.24; Oxford Nanopore Technologies, Oxford, UK) was used following the “Ligation sequencing amplicons” protocol, according to the manufacturer’s instructions with no DNA Control Sample. In brief, a mix of the three PCR pools was used as input (12.5 μl) for End-prep. After this, the DNA concentration for each sample was determined and the input to the next step (Native barcoding ligation) was normalized between samples. Either 130 ng of DNA per sample was used, or the input for all samples were normalized to the sample with the lowest concentration (not below 7.5 ng DNA in total). The final library was diluted to 20 fmol.

### Amplicon sequencing

Sequencing was performed on a MinION device using R10.4.1 flow cells (FLO-MIN114; Oxford Nanopore Technologies, UK). The flow cells were prepared according to the manufacturer’s instructions and between 5 and 24 barcoded samples were included per run. Depending on the remaining viable pore count, flow cells were washed and reused to sequence additional samples. Basecalling was performed with Guppy v6.4.6 using the high accuracy basecalling model. Sequencing coverage of each amplicon and sample was monitored throughout the runs using the Artic Network RAMPART tool (https://artic-network.github.io/rampart/) with a custom protocol.

### WGS

Previously acquired WGS data were used for validation when available. Isolates grown from respective samples were generated as a part of routine diagnosis and surveillance at NRL-TB, Portuguese NIH, and WGS was performed as described previously [16]. In brief, samples were subjected to Nextera XT library preparation and paired-end sequencing on an Illumina MiSeq or NextSeq apparatus (Illumina, San Diego, CA, USA).

### Sequence data analysis and gDST

Nanopore sequencing reads were subjected to quality control and improvement using Porechop (https://github.com/rrwick/Porechop) for adapter and barcode trimming and NanoFilt (https://github.com/wdecoster/nanofilt) to filter out reads with <500 bp and an average read quality score <10, and trim 50 bp on each end to remove primer regions. To identify potential contaminations, Kraken2 v2.0.7-beta [17] (https://github.com/DerrickWood/kraken2) was used for taxonomical classification of reads with the Standard-16 16 GB database (26 September 2022, available at: https://benlangmead.github.io/aws-indexes/k2). To assess the targeted sequencing success, the sequencing reads were mapped against a multi-fasta reference file with all the targeted genomic positions (amplicons), excluding the primers flanking regions (50 bp) in each amplicon, using minimap2 [18] (https://github.com/lh3/minimap2), and the depth of coverage was assessed with SAMtools [19] (https://github.com/samtools/samtools).

For both WGS and tNGS data, gDST and variant calling were performed with TB-profiler v6.2.0 using raw reads (tNGS) and the default settings [3] (webserver; https://tbdr.lshtm.ac.uk). These results were then further classified as valid (S/R) or undetermined (U) using an in-house analysis script based on the depth of coverage after quality control for all positions of interest (POI) relevant to the tested antibiotic. POIs included all mutations covered by our amplicons being listed in the TB Profiler database (https://github.com/jodyphelan/TBProfiler), including WHO class I and class II mutations [9] minus deletions. For amino acid substitutions, all three nucleotides were considered. A depth of coverage of ≥10x across all POIs was classified as valid, while coverage below this at a single POI for that antibiotic was classified as undetermined.

### Data analysis and statistics

Data analysis and statistics were performed using R v.4.3.2 with R Studio 2024.09.0 Build 375 and GraphPad Prism v.10.3.0. Sensitivity and specificity, including 95% confidence intervals, were calculated using the epiR R-package v.2.0.74. An ordinary one-way ANOVA using Turkey method for adjusting for multiple comparisons was used for comparing different smear positivity and an unpaired t-test for comparing sample types.

### Ethical statement

This research complies with all relevant ethical regulations. The study used clinical samples previously processed by the Portuguese NIH NRL-TB for diagnostic purposes and antibiotic resistance detection, following formal medical requests issued by hospital clinicians. Informed consent for these clinical procedures is obtained exclusively at the hospital level, and the Portuguese NIH is not involved in this process nor has access to the consent forms; it receives only the clinical requisition. In the present study, the accuracy assessment of the assay developed by the NRL involved manipulating the samples strictly for the same purpose as the original clinician request: TB detection and resistance prediction, thus requiring no additional permissions.

## Results

### Amplicon design and antibiotic targets

**Figure 1.**
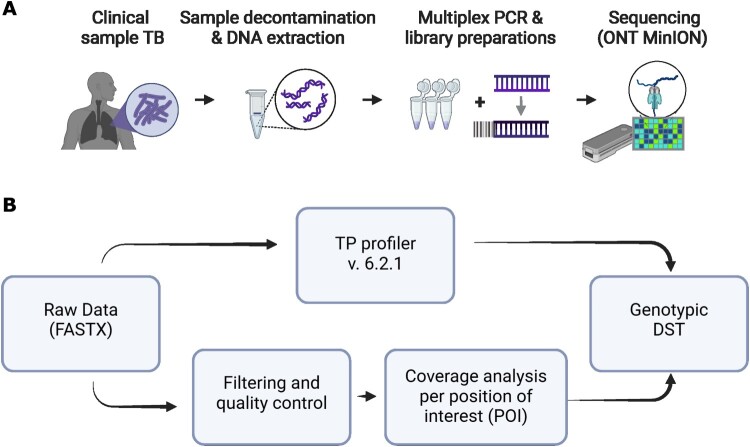
Graphic summary of the workflow. Laboratory (A) and bioinformatics (B) workflow of tNGS of *M. tuberculosis* for genotypic drug-susceptibility testing (DST) directly from clinical samples from pulmonary TB patients. Illustration generated using biorender.com.

We developed an amplicon-based targeted sequencing approach targeting 19 resistance-associated genes/regions that are known to be associated with resistance against 20 antibiotics and evaluated it on 71 clinical samples from patients with confirmed pulmonary TB (Table 1, Figure 1(A)). For gDST determination, the open-source pipeline TB-Profiler [3] was used in parallel with a tailored bioinformatics analysis to complement the results, aiding the validation of the protocol and interpretation of the results (Figure 1(B)).

**Table 1.** Antibiotics and amplicons for tNGS.

Antibiotics	Code	Gene(s)	Amplicons per gene	Gene coverage
**First line**				
Rifampicin	RIF	rpoB	2	373–1754
Isoniazid	INH	inhA / katG	2 / 5	−892–499 / – 115–3346
Ethambutol	EMB	embB	1	857–1617
Pyrazinamide	PZA	pncA	2	−249–1575
**Second line/aminoglycosides**				
Amikacin	AMK	rrs / eis	2 /1	−151–1537 / – 199–764
Kanamycin	KAN	rrs / eis	2 /1	−151–1537 / – 199–764
Capreomycin	CAP	rrs / tlyA	2 /1	−151–1537 / – 149–807
**Fluoroquinolones**				
Moxifloxacin/Levofloxacin	MFX/LFX	gyrA / gyrB	1 / 1	−154–887 / 1145–2028
Ofloxacin	OFX	gyrA / gyrB	1 / 1	−154–887 / 1145–2028
Ciprofloxacin	CIP	gyrA / gyrB	1 / 1	−154–887/1145–2028
**Others**				
Cycloserine	CYCLO	ald^a^/ alr^a^	2 / 2	−161–1116/–174–1227
Ethionamide/Prothionamide	ETH/PTH	ethA / inhA	2 / 2	−168–1470/–892–499
Clofazimine/Bedaquiline	CFZ/BDQ	Rv0678	1	−251–498
Delamanid/Pretomanid	DLM/PMD	ddn	1	−369–456
Para-aminosalicylic_acid	PAS	folC^a^ / thyA^a^	1 / 1	−329–716/–145–792
Linezolid	LZD	rplC / rrl	1 / 1	12–654/2005–2952

Note: Genes of interest, amplicons and gene coverage of the tNGS panel. Gene coverage is relative to the start of the coding region of the respective gene in reference genome H37Rv (accession number NC_000962.3).

### Clinical samples and sequencing

Samples were collected as a part of routine diagnosis at the NRL-TB, Portuguese NIH and included both sputum samples and other sample types of different smear positivity/AFB scoring, 0–3+ (Table 2). A total of 20 (28%) samples displayed some level of phenotypic drug resistance, with resistance to Isoniazid (INH) being the most common. Four sputum samples were MDR (resistant to INH and RIF), for which an extended panel of phenotypic DST was performed, including 16 additional antibiotics (see materials and methods).

**Table 2.** Clinical samples included in the study categorized by sample type and smear positivity.

					Phenotypic resistance of isolate							
Sample type	Smear positivity	No. of samples	WGS		INH		PZA		EMB		RIF	
			N	%	N	%	N	%	N	%	N	%
Sputum	0	12	6	50%	2	17%	0		0		0	
	1	18	15	83%	5	28%	0		1	6%	2	11%
	2	11	8	73%	5	46%	1	9%	1	9%	1	9%
	3	12	9	69%	5	42%	1	8%	1	8%	1	8%
Other^a^	1	4	4	100%	1	25%	0		0		0	
	2	4	3	75%	1	25%	0		0		0	
	3	10	8	89%	1	10%	0		0		0	
**Total**		**71**	**53**	**75%**	**20**	**28%**	**2**	**3%**	**3**	**4%**	**4**	**6%**

Note: The availability of whole genome sequencing (WGS) data and phenotypic drug resistance results for the corresponding isolates is described.

^a^ Bronchial secretions (n = 10), bronchial lavage (n = 3), respiratory secretions (n = 1), respiratory secretions aspirate (n = 1), bronchoalveolar lavage (n = 1) and tracheal aspirate (n = 1).

Between 18,322 and 351,046 (mean 128,503) reads were generated per sample, with on-target *M. tuberculosis* reads ranging from 0.3 to 99.2%. The average depth of each amplicon obtained from the tNGS ranged from zero to 61,410x. Of all amplicons, 75% (1,562) were sequenced to >100x depth of coverage at all positions, 84% (1,751) to >30x depth of coverage and 91% (1,895) to >10x depth of coverage. The median proportion of amplicons with full coverage was 97% at >30x and 100% at >10x depth of coverage; 33/71 samples (46%) had full coverage of all amplicons at >30x depth of coverage and 43/71 samples (61%) at >10x depth of coverage.

### High concordance between tNGS results and pDST

We started comparing the gDST results from the tNGS approach with the pDST profile for the first line antibiotics INH, PZA, EMB and RIF. Using our tNGS approach, 248 antibiotic profiles would be virtually expected, where an antibiotic profile was defined as the result (resistant-R, sensitive-S or undetermined-U) for a single antibiotic for a single sample. From this, we captured 224 (90%), while the remaining 24 (10%) profiles were categorized as undetermined (here defined as below 10x depth of coverage in at least one POI for that antibiotic) (Table 3).

**Table 3.** Comparison of genotypic and phenotypic DST results for first-line antibiotics.

	pDST resistant				pDST susceptible							
		tNGS results				tNGS results			CA total (%)	CA^a^ (%)	Sensitivity^a^ [95 CI]	Specificity^a^ [95 CI]
Antibiotic	Total	R	S	U	Total	R	S	U				
INH	20	15	3	2	51	2	40	9	77.5	91.7	0.83 [0.59, 0.96]	0.95 [0.84, 0.99]
PZA	2	2	0	0	69	0	67	2	97.2	100	1.0 [0.16, 1]	1.0 [0.95, 1]
EMB	3	2	1	0	32	0	32	0	97.1	97.1	0.67 [0.19, 0.99]	0.97 [0.89, 1]
RIF	4	4	0	0	67	0	56	11	84.5	100	1.0 [0.40, 1]	1.0 [0.94, 1]
**Total**	**29**	**23**	**4**	**2**	**219**	**2**	**195**	**22**	**87.9**	**97.3**	**0.85 [0.66, 0.96]**	**0.99 [0.96, 1]**

Note: Genotypic DST from tNGS was compared to phenotypic results for all samples (n = 71). Categorical agreement (CA) was calculated with (total) and without undetermined results, defined as below 10x depth of coverage in at least one POI for that antibiotic. The 95% confidence interval (95 CI) for sensitivity and specificity is displayed in brackets.

^a^ Calculated excluding undetermined results.

While our conservative categorization approach classified two INH resistant samples as undetermined by tNGS, these were still correctly classified as resistant by TB profiler based on the partial data available. For 218 profiles, there was a match between the gDST and pDST results, leading to a categorical agreement of 88% for all profiles and 97% excluding undetermined results.

The categorical agreement between tNGS-gDST and pDST for the individual antibiotics, both including and excluding undetermined results, was as follows: INH (78%/92%), PZA (97%/100%), EMB (97%/97%), and RIF (85%/100%) (Table 3). Adjusting the threshold for undetermined results to below 30x depth of coverage increased the number of undetermined profiles to 40 (16%), resulting in an overall categorical agreement of 82% with only minor improvements in sensitivity and specificity (Table S3).

For the four MDR isolates, comprising 48 antibiotic profiles, the tNGS results were compared to pDST for the extended panel of antibiotics (Table S4) with an overall CA of 89%, or 93% excluding undetermined results (a single profile). Among resistant phenotypes (first- and second-line antibiotics), five were classified by tNGS as susceptible (INH, n = 3; EMB, n = 1; CYCLO, n = 1) (Table 3, Table S4). In contrast, gDST classified two samples as resistant to INH and a third as resistant to MFX/LFX, AMK and CAP, despite these exhibiting susceptible phenotypes. Among these, one of the INH profiles (sample ID: MTB-PT-tNGS-10) and for all MFX/LFX profiles (sample IDs: MTB-PT-tNGS-6a/65), the identified mutations are described to provide low-level resistance, *inhA* p.Ser94Ala and *gyrA* p.Asp94Ala, respectively (Table S2). The sample classified as resistant to AMK and CAP by tNGS (sample ID: MTB-PT-tNGS-06a) had the *rrs* n.1401A > G mutation, known to confer resistance to aminoglycosides (AMK, CAP and KAN) [20], though only KAN resistance was observed phenotypically (Table S2), highlighting the added information value of gDST.

### Resistance mutations identified by tNGS closely match those found by WGS

For samples with available WGS data (n = 53), we compared the gDST results from tNGS and WGS for all antibiotics included in TB profiler, totally 822 antibiotic profiles. Our tNGS approach correctly classified 756 profiles, with four profiles misclassified compared to WGS results and 62 (8%) profiles undetermined, resulting in an overall categorical agreement of 92% and 99.5% when excluding undetermined profiles (Table 4). Importantly, the CAP resistant sample, that was classified as undetermined by tNGS (sample ID: MTB-PT-tNGS-31), was correctly classified as resistant by TB profiler based on partial tNGS data, showing the added value also of partial sequencing data.

**Table 4.** Comparison of genotypic DST, tNGS and WGS for all antibiotics.

	WGS resistant				WGS susceptible							
		tNGS results			tNGS results				CA total (%)	CA^a^ (%)	Sensitivity^a^ [95 CI]	Specificity^a^ [95 CI]
Antibiotic	Total	R	S	U	Total	R	S	U				
INH	12	12	0	0	41	0	33	8	84.9	100	1.0 [0.74, 1]	1.0 [0.89, 1]
PZA	2	2	0	0	51	0	51	0	100	100	1.0 [0.16, 1]	1.0 [0.93, 1]
EMB	3	2	1	0	24	0	24	0	96.3	96.3	0.67 [0.09, 0.99]	1.0 [0.86,1]
RIF	4	4	0	0	49	0	40	9	83	100	1.0 [0.40, 1]	1.0 [0.91, 1]
MFX	1	1	0	0	52	0	49	3	94.3	100	1.0 [0.03, 1]	1.0 [0.93, 1]
LFX	1	1	0	0	52	0	49	3	94.3	100	1.0 [0.03, 1]	1.0 [0.93, 1]
BDQ	0	0	0	0	53	0	51	2	96.2	100	NA	1.0 [0.93, 1]
DLM	0	0	0	0	53	0	51	2	96.2	100	NA	1.0 [0.92, 1]
LZD	0	0	0	0	53	0	47	6	88.7	100	NA	1.0 [0.93, 1]
AMK	0	0	0	0	53	1	50	2	94.3	98	NA	0.98 [0.90 1]
KAN	0	0	0	0	53	1	50	2	94.3	98	NA	0.98 [0.90, 1]
CAP	1	0	0	1	52	1	48	3	90.6	98	NA	0.98 [0.89, 1]
CFZ	0	0	0	0	53	0	51	2	96.2	100	NA	1.0 [0.94, 1]
ETH	4	4	0	0	49	0	40	9	83	100	1.0 [0.40, 1]	1.0 [0.91, 1]
PAS	1	0	0	1	52	0	49	3	92.5	100	NA	1.0 [0.93, 1]
CYCLO	0	0	0	0	53	0	47	6	88.7	100	NA	1.0 [0.92, 1]
**Total**	**29**	**26**	**1**	**2**	**793**	**3**	**730**	**60**	**92**	**99.5**	**0.96 [0.81, 1]**	**1.0 [0.99, 1]**

Note: Genotypic DST results obtained using tNGS were compared to genotypic DST results using whole genome sequencing (WGS) data from corresponding isolates, when available (n = 53). Categorical agreement (CA) was calculated with (total) and without undetermined results, defined as below 10x depth of coverage in at least one POI for that antibiotic.

^a^ Calculated excluding undetermined results.

Adjusting the threshold for undetermined results to below 30x depth of coverage increased the number of undetermined profiles from 8% to 15% (127), missing two INH resistant profiles. This resulted in a categorical agreement of 84% for all profiles, with no decrease of discordant profiles or apparent gain in specificity (Table S5).

For the 11 profiles with a discordant phenotype and tNGS results, eight had WGS data. For five profiles (INH = 2, CYCLO = 1, MFX = 1, LFX = 1), the WGS results aligned with the tNGS. For the remaining three profiles, the resistance mutation identified by tNGS was also observed in the WGS data but did not pass the quality control (AMK = 1, KAN = 1; *rrs* n.1401A > G, sample ID: MTB-PT-tNGS-06a) or the main resistance conferring mutation identified by WGS was in a gene not covered by our panel (EMB = 1; *embA*, sample ID: MTB-PT-tNGS-53, Table S2). For this sample, WGS also showed a low-prevalence mutation in *embB* (p.Met306Val), which was observed in the tNGS data upon manual inspection (15% of reads, >14,000 depth of coverage).

### Detection of non-resistance associated mutations

To further validate our results, we analysed mutations not associated with resistance in the regions covered by our panel. In total, WGS identified 260 mutations across 41 unique positions (Table S5). Our tNGS approach correctly identified 231 (89%) mutations, while 14 (5%) were not identified by tNGS and 15 (6%) positions remained undetermined (Table S6). Raising the threshold to >30x depth of coverage reduced the number of correctly identified mutations to 218 (84%), with 12 (5%) mutations not detected, while doubling 30 (12%) the undetermined positions (Table S6). Twelve of the 13 additional mutations identified with a 10x threshold were in mutation hot-spots (defined as positions where >50% of samples presented mutations), suggesting that these are likely not the result of random sequencing errors.

**Figure 2.**
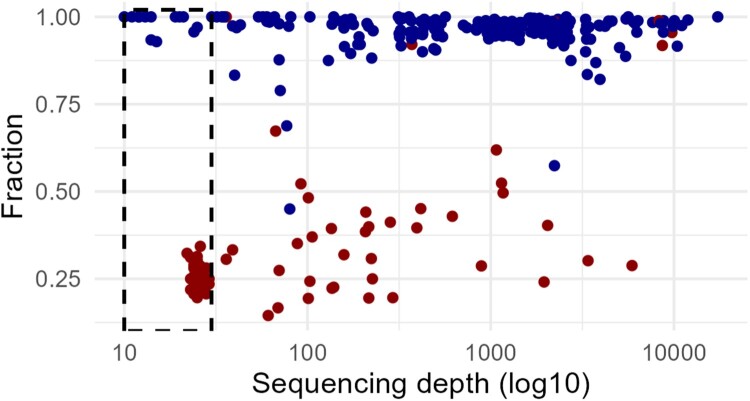
Evaluation of mutations not associated with resistance. Sequencing depth and fraction (proportion of reads with mutation) of mutations identified by both WGS and tNGS (blue) or only tNGS (red). Dotted box represents mutations excluded by using 30x as threshold for undetermined results.

We identified 95 mutations by tNGS that were not present in the WGS data (possible false positive results) at a threshold of 10x. To explore these further, we compared sequencing depth and sequencing fraction for true positives (mutations identified by both methods, n = 231) and possible false positives (tNGS-unique mutations). We found that the 53 tNGS-unique mutations only identified with the lower threshold (10x) were found in a single sample with a low proportion of reads with the mutation (Figure 2). This sample had a low proportion of *M. tuberculosis* reads (2%) and all mutations were found in *gyrA* and *rrs*, two genes with known homology to other bacterial species. Together, this suggests low-level contamination in a single sample with poor amplification, rather than sequencing error.

In contrast to mutations identified by both methods (true positives), 27 of the remaining 42 tNGS-unique mutations (30x threshold) presented in relatively low fraction (<75% of reads) with adequate sequencing depth (>100x) (Figure 2). These 27 mutations were identified in 14 different genes from nine samples, suggesting that they could represent genetic heterogeneity selected against during culture.

### Sequencing coverage is not strongly influenced by sample type or smear positivity level

**Figure 3.**
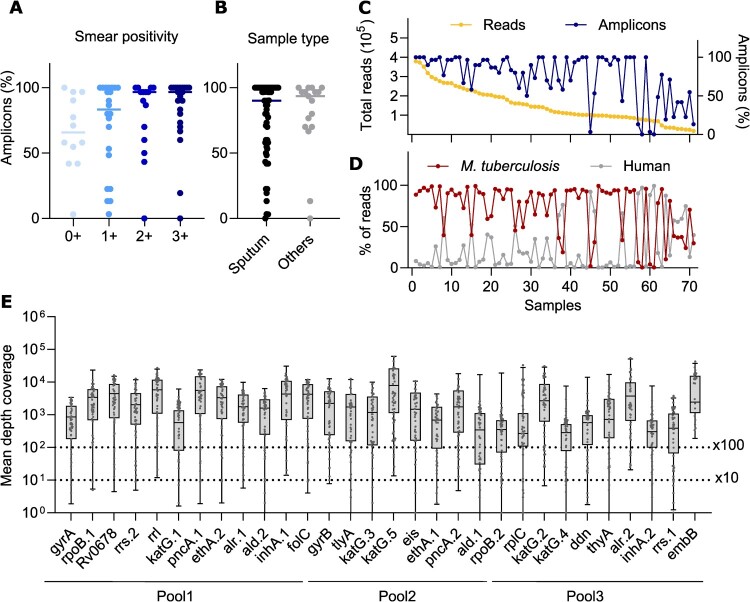
Evaluation of factors potentially influencing sequencing coverage. Proportion of amplicons with >100x sequencing depth at all positions according to (A) smear positivity and (B) sample type. (C) Comparison of the number of sequencing reads (yellow) and proportion of amplicons with >100x sequencing depth at all positions (blue) for each sample. (D) percentage of reads corresponding to the *M. tuberculosis* (red) or human (grey) genome, for each sample. (E) Mean sequencing depth of coverage per amplicon. All samples are shown as individual dots (n = 71).

To assess factors affecting sequencing coverage, we analysed amplicons with >100x depth across sample types and smear positivity levels. While higher smear positivity showed a trend toward increased coverage (Figure 3(A)), the difference was not significant (0 + vs. 3+, *p* = 0.418). Similarly, no significant difference was observed between sputum and other sample types (*p* = 0.543, Figure 3(B)), although the low number of each non-sputum type restricts interpretability. These findings suggest that neither smear positivity nor sample type alone explains the variability in sequencing performance, although a larger sample set may be needed for a definite evaluation.

Samples with higher total number of reads generally had a higher proportion of amplicons with >100x depth (Figure 3(C)). Among the samples, 73% (n = 58) had >50% of mapped reads corresponding to *M. tuberculosis*. Human DNA was the main contaminant (Figure 3(D)), with minimal non-specific bacterial/viral amplification. This is important given the homology of *rrs*, *rrl*, *rpoB*, and *gyrA/B* with other bacteria. Only 3 samples had >10% (17–33%) non-human/*M. tuberculosis* reads, primarily identified as *Prevotella* (n = 1), *Pseudomonas* (n = 1), and *Streptococcus* (n = 1), although without any apparent impact on the gDST results.

We also analysed the mean depth of coverage across amplicons. Generally, the amplicons in pool 3 showed slightly lower mean depth of coverage (Figure 3(E)), despite the visual classification of bands on the agar gel used for determining pooling ratios did not differ systematically between the pools. Still, all but four amplicons (*katG.4, ald.1, rpoB.2* and *rrs.1*) were covered at >100x average depth of coverage for >75% of all samples.

### Time and cost requirements

When a sample is received at the Portuguese NRL-TB it is decontaminated and used as input for smear microscopy and molecular diagnosis within 24–48 h. Our tNGS approach uses the remaining sample followed by DNA extraction and multiplex PCR (1.5 days, hands-on approx. 2 h), library preparation (1 day, hands-on approx. 2 h) and lastly sequencing and analysis (1 day, hands-on approx. 2 h). Taken together, results for tNGS-based gDST for the full panel of 20 antibiotics may be available in five days after the sample reception. This highly contrasts the time required for pDST and WGS-based gDST, that is dependent on the growth rate of the *M. tuberculosis* strain and may take between 2 and 15 weeks. The current estimated cost of this tNGS approach is roughly 40–100€ per sample, depending on the NGS platform and number of samples per run. In comparison, WGS costs per sample are approx. 250€, and the estimated cost of pDST may go up to 40–70€ for the complete set of antibiotics.

## Discussion

We designed an amplicon-based targeted sequencing approach for *M. tuberculosis* and evaluated it directly on clinical samples from patients with confirmed pulmonary TB. We demonstrate high concordance with pDST and WGS (categorical agreement of 97 to >99% depending on reference method), while significantly reducing the time to results and associated costs. In addition, the flexibility of this methodology allows for a dynamic adaptation to new genetic antimicrobial resistance markers. While we chose ONT technology (MinION) for the sequencing due to its versatility and speed, the amplicon design is compatible with other sequencing technologies, such as Illumina. The entire workflow can be completed within five days, providing significantly reduced turnaround time compared to traditional culture-based methods.

Compared to previous studies or commercial kits currently available [21–26], our study presents one of the far most comprehensive panels, especially among those that have been validated directly on clinical samples [21–23]. Our panel targets a broad range of antibiotics, also including second-line antibiotics and newer agents such as DLM/PMD, reflecting the evolving landscape of drug resistance and treatment regimens. Target selection for the panel was based on the WHO updated 2023 catalogue [9]. According to this, 14 genes or regions (*gyrA, gyrB, ropB, Rv0678, rpsL, rrs, inhA, tlyA, katG, pncA, eis, embB, ethA, gid*) may harbour class I mutations (associated with resistance) relevant for resistance to INH, RIF, EMB, PZA, MFX/LFX, CIP, BDQ/CFZ, AMK, CAP, KAN, ETH/PTO. We also included two genes (*rrl, ddn*) containing class II mutations (associated with intermediate resistance) to also capture the most relevant regions for LZD and DLM/PMD resistance. Four other genes (*alr, ald, folC, thyA*) included in the TB Profiler database were then added based on substantial evidence of resistance mutations for CYCLO [27,28] and PAS [29,30]. While streptomycin was not included in our analysis due to its current limited therapeutic use, additional amplicons covering e.g. *rpsL* and *gid* could be added to the panel if deemed relevant in the local context [9]. Beyond streptomycin, the panel could be expanded to incorporate additional targets as our understanding of resistance mechanisms evolve, examples of potential targets of interest include *thyX* (PAS) [31], *atpE* (BDQ/CFZ), or *fbiA/B/C* (DLM/PMD) [9]. Such additions would allow the panel to adapt to emerging resistance mutations and national priorities.

Thorough design and several rounds of optimization were made to ensure good coverage. Most primer pairs performed well in individual PCRs and later in multiplex pools, despite the known challenge of GC-rich regions of the *M. tuberculosis* genome. However, as this work was carried out with limited resources as part of in-house diagnostic development, there is potential for further optimization if the set of targets is expanded or adapted.

In total, five of 33 phenotypically resistant profiles were classified as sensitive by tNGS, namely, INH (n = 3), CYCLO (n = 1) and EMB (n = 1). For CYCLO and the one INH resistant profile with WGS data available, our tNGS results are in line with the WGS results for the corresponding isolates. Discordance between phenotypic and genotypic DST have been reported previously, in particular for INH, and likely suggests uncharacterized resistance mechanisms [32,33].

The sample classified as EMB-susceptible by tNGS was identified as resistant by WGS in line with the phenotypic results based on two mutations: *embB*:p.Met306Val (17% of reads) and *embA*:c.−16C > G (82% of reads). While the intergenic region of *embC-embA* has been implicated to be important for resistance against EMB [34], the WHO 2023 catalogue does not classify any *embA* mutations as associated with resistance [9]. As the isolate was phenotypically EMB-resistant, *embA* could be considered in an expanded panel. The low prevalence mutation in *embB* for this isolate was observed also in the tNGS data upon manual inspection and would have been picked up if present at a higher proportion.

A threshold of <10x at a single POI for a given antibiotic was used to define an undetermined result, which is also the default cutoff for the command line of TB profiler [17]. Given the error-prone nature of ONT sequencing, we also performed the analysis with the more conservative >30x threshold. Our results do not show clear signs of sequencing errors affecting the results. Rather, the two additional pDST-tNGS discordant profiles, detected at 10x but not at 30x, had WGS data results in line with the tNGS-data, and the increased number of mutations not associated with resistance detected solely by tNGS likely represents low-level bacterial contamination in a single sample with poor amplification. However, direct validation of the sequencing platform as performed in other studies [35,36], would be required to fully exclude potential platform-specific biases. Interestingly, we could see that even when a tNGS profile was classified as undetermined, TB profiler successfully identified three of the four resistant pDST/WGS profiles (Table S2). This highlights the added value also of partial sequencing data.

Among the limitations of the study, we highlight that only four samples included a pDST profile beyond the first line antibiotics, and WGS data were only available for 75% of samples, restricting the insights into some of the pDST-gDST discrepancies. The number of specimens per sample type was small and constrained by clinical availability, limiting robust conclusions for individual non-sputum sample types. Similarly, no smear-negative samples were included in our study, although they are known to contribute to TB transmission [37] and targeted sequencing from such samples has been shown to be feasible [21]. Also, while we did not observe clear differences in sequencing coverage between the collection years (2020–2024), the potential negative effects of long-term storage cannot be entirely excluded. Additionally, we would have benefitted from more samples with diverse resistant phenotypes, which would allow a more comprehensive validation of our approach in capturing rare resistance mutations. To partially compensate for this, we compared the tNGS results with the respective WGS data in regards to mutations not associated with resistance. This approach showed that our tNGS method identified 89% of mutations in the target regions, suggesting that, if resistance mutations would have been present, we would have captured these to a high extent. However, comparing WGS data from isolates to tNGS results directly from clinical samples may not be completely straightforward, and it must be taken into consideration that mutations may be acquired/enriched or lost/selected against during culture. Lastly, this study is limited by the absence of pDST for the newer agents BDQ, DLM, and PMD. During the study period, these assays were not available in Portugal, and standardized reference methods were not yet routinely implemented internationally. Furthermore, resistance mechanisms for these agents, particularly for PMD and DLM, are still being elucidated, limiting the interpretive value of both phenotypic and genotypic data. Future investigations should aim to integrate validated pDST and genomic data for these drugs to support more robust benchmarking of molecular resistance prediction tools.

## Conclusion

The tNGS approach developed in this study demonstrates a rapid, flexible, and cost-effective method for genotypic DST directly from clinical samples, providing a valuable complement or alternative to traditional phenotypic testing. This approach shows high concordance with pDST and WGS from corresponding isolates and provides the ability to detect low-level resistance mutations in clinical samples possibly lost during culturing. Our suggested approach supports decentralization of DST by its compatibility with compact sequencing devices (e.g. MinION) and use of webservers with reduced need for bioinformatics expertise during routine analysis (e.g. TB Profiler). By sharing primer sequences and detailed reaction conditions and optimization steps, we hope to facilitate uptake, enable local target adaptation and further community-driven optimization. This flexibility supports decentralization in low-incidence settings with limited BSL-3 infrastructure for culture, and offers a feasible alternative to phenotypic testing in high-burden and resource-limited settings, ultimately contributing to the global TB eradication efforts.

## Supplementary Material

Supplementary_TB_TargetedSeq_Rosendal_revised.docx

TableS2_Individual_samples_list_revised.xlsx

## Access to data

The sequencing reads generated during this study (reads mapping against target regions) are deposited in the European Nucleotide Archive [Bioproject accession number PRJEB88154].

## References

[1] WHO. Tuberculosis. WHO; 2024 [cited 2025 Mar 1]. Available from: https://www.who.int/news-room/fact-sheets/detail/tuberculosis

[2] Liebenberg D, Gordhan BG, Kana BD. Drug resistant tuberculosis: implications for transmission, diagnosis, and disease management. Front Cell Infect Microbiol. 2022;12:943545. doi:10.3389/fcimb.2022.943545 36211964 PMC9538507

[3] Phelan JE, O’Sullivan DM, Machado D, et al. Integrating informatics tools and portable sequencing technology for rapid detection of resistance to anti-tuberculous drugs. Genome Med. 2019;11(1):41. doi:10.1186/s13073-019-0650-x 31234910 PMC6591855

[4] Satta G, Lipman M, Smith GP, et al. Mycobacterium tuberculosis and whole-genome sequencing: how close are we to unleashing its full potential? Clin Microbiol Infect. 2018;24(6):604–609. doi:10.1016/j.cmi.2017.10.030 29108952

[5] Brown AC, Bryant JM, Einer-Jensen K, et al. Rapid whole-genome sequencing of Mycobacterium tuberculosis isolates directly from clinical samples. J Clin Microbiol. 2015;53(7):2230–2237. doi:10.1128/JCM.00486-15 25972414 PMC4473240

[6] Macedo R, Isidro J, Ferreira R, et al. Molecular capture of Mycobacterium tuberculosis genomes directly from clinical samples: a potential backup approach for epidemiological and drug susceptibility inferences. Int J Mol Sci. 2023;24(3):2912. doi:10.3390/ijms24032912 36769230 PMC9918089

[7] MacLean E, Kohli M, Weber SF, et al. Advances in molecular diagnosis of tuberculosis. J Clin Microbiol. 2020;58(10). doi:10.1128/JCM.01582-19 PMC7512154 32759357

[8] Nandlal L, Perumal R, Naidoo K. Rapid molecular assays for the diagnosis of drug-resistant tuberculosis. Infect Drug Resist. 2022;15:4971–4984. doi:10.2147/IDR.S381643 36060232 PMC9438776

[9] GTB. Catalogue of mutations in Mycobacterium tuberculosis complex and their association with drug resistance. WHO, editor. Geneva: WHO; 2023.

[10] Dippenaar A, Goossens SN, Grobbelaar M, et al. Nanopore sequencing for Mycobacterium tuberculosis: a critical review of the literature, new developments, and future opportunities. J Clin Microbiol. 2022;60(1):e0064621. doi:10.1128/JCM.00646-21 34133895 PMC8769739

[11] Schwab TC, Perrig L, Göller PC, et al. Targeted next-generation sequencing to diagnose drug-resistant tuberculosis: a systematic review and meta-analysis. Lancet Infect Dis. 2024;24(10):1162–1176. doi:10.1016/S1473-3099(24)00263-9 38795712 PMC11881551

[12] GTB. Who consolidated guidelines on tuberculosis Module 1: prevention – tuberculosis preventive treatment. 2nd ed. Geneva: World Health Organization; 2024. p. 268. 39298638

[13] Rozen S, Skaletsky H. Primer3 on the WWW for general users and for biologist programmers. Methods Mol Biol. 2000;132:365–386. 10547847 10.1385/1-59259-192-2:365

[14] Perez G, Barber G, Benet-Pages A, et al. The UCSC genome browser database: 2025 update. Nucleic Acids Res. 2025;53(D1):D1243–D1249. doi:10.1093/nar/gkae974 39460617 PMC11701590

[15] Camacho C, Coulouris G, Avagyan V, et al. Blast+: architecture and applications. BMC Bioinformatics. 2009;10:421. doi:10.1186/1471-2105-10-421 20003500 PMC2803857

[16] Macedo R, Pinto M, Borges V, et al. Evaluation of a gene-by-gene approach for prospective whole-genome sequencing-based surveillance of multidrug resistant Mycobacterium tuberculosis. Tuberculosis (Edinb). 2019;115:81–88. doi:10.1016/j.tube.2019.02.006 30948181

[17] Wood DE, Lu J, Langmead B. Improved metagenomic analysis with Kraken 2. Genome Biol. 2019;20(1):257. doi:10.1186/s13059-019-1891-0 31779668 PMC6883579

[18] Li H. Minimap2: pairwise alignment for nucleotide sequences. Bioinformatics. 2018;34(18):3094–3100. doi:10.1093/bioinformatics/bty191 29750242 PMC6137996

[19] Danecek P, Bonfield JK, Liddle J, et al. Twelve years of SAMtools and BCFtools. Gigascience. 2021;10(2). doi:10.1093/gigascience/giab008 PMC7931819 33590861

[20] Du Q, Dai G, Long Q, et al. Mycobacterium tuberculosis rrs A1401G mutation correlates with high-level resistance to kanamycin, amikacin, and capreomycin in clinical isolates from mainland China. Diagn Microbiol Infect Dis. 2013;77(2):138–142. doi:10.1016/j.diagmicrobio.2013.06.031 23948547

[21] Chan WS, Au CH, Chung Y, et al. Rapid and economical drug resistance profiling with Nanopore MinION for clinical specimens with low bacillary burden of Mycobacterium tuberculosis. BMC Res Notes. 2020;13(1):444. doi:10.1186/s13104-020-05287-9 32948225 PMC7501614

[22] Colman RE, Anderson J, Lemmer D, et al. Rapid drug susceptibility testing of drug-resistant Mycobacterium tuberculosis isolates directly from clinical samples by use of amplicon sequencing: a proof-of-concept study. J Clin Microbiol. 2016;54(8):2058–2067. doi:10.1128/JCM.00535-16 27225403 PMC4963505

[23] Murphy SG, Smith C, Lapierre P, et al. Direct detection of drug-resistant Mycobacterium tuberculosis using targeted next generation sequencing. Front Public Health. 2023;11:1206056. doi:10.3389/fpubh.2023.1206056 37457262 PMC10340549

[24] Tafess K, Ng TTL, Lao HY, et al. Targeted-Sequencing workflows for comprehensive drug resistance profiling of Mycobacterium tuberculosis cultures using two commercial sequencing platforms: comparison of analytical and diagnostic performance, turnaround time, and cost. Clin Chem. 2020;66(6):809–820. doi:10.1093/clinchem/hvaa092 32402055

[25] Wu SH, Xiao Y-X, Hsiao H-C, et al. Development and assessment of a novel whole-gene-based targeted next-generation sequencing assay for detecting the susceptibility of mycobacterium tuberculosis to 14 drugs. Microbiol Spectr. 2022;10(6):e0260522. doi:10.1128/spectrum.02605-22 36255328 PMC9769975

[26] Carandang T, Cunanan DJ, Co GS, et al. Diagnostic accuracy of Nanopore sequencing for detecting Mycobacterium tuberculosis and drug-resistant strains: a systematic review and meta-analysis. Sci Rep. 2025;15(1):11626. doi:10.1038/s41598-025-90089-x 40185766 PMC11971303

[27] Desjardins CA, Cohen KA, Munsamy V, et al. Genomic and functional analyses of Mycobacterium tuberculosis strains implicate ald in D-cycloserine resistance. Nat Genet. 2016;48(5):544–551. doi:10.1038/ng.3548 27064254 PMC4848111

[28] Nakatani Y, Opel-Reading HK, Merker M, et al. Role of alanine racemase mutations in Mycobacterium tuberculosis d-cycloserine resistance. Antimicrob Agents Chemother. 2017;61(12):e01575–17. doi:10.1128/AAC.01575-17 PMC5700341 28971867

[29] Luo M, Li K, Zhang H, et al. Molecular characterization of para-aminosalicylic acid resistant Mycobacterium tuberculosis clinical isolates in southwestern China. Infect Drug Resist. 2019;12:2269–2275. doi:10.2147/IDR.S207259 31440065 PMC6664864

[30] Rengarajan J, Sassetti CM, Naroditskaya V, et al. The folate pathway is a target for resistance to the drug para-aminosalicylic acid (PAS) in mycobacteria. Mol Microbiol. 2004;53(1):275–282. doi:10.1111/j.1365-2958.2004.04120.x 15225321

[31] Zeng R, He L, Zhang B, et al. Association between mutations in a thyX-hsdS.1 region and para-aminosalicylic acid resistance in Mycobacterium tuberculosis clinical isolates. Emerg Microbes Infect. 2023;12(2):2276339. doi:10.1080/22221751.2023.2276339 38029724 PMC10769527

[32] Bonsa Z, Tadesse M, Balay G, et al. Discordance between genotypic and phenotypic methods for the detection of rifampicin and isoniazid resistant Mycobacterium tuberculosis and the correlation with patient treatment outcomes. J Clin Tuberc Other Mycobact Dis. 2024;34:100410. doi:10.1016/j.jctube.2023.100410 38225941 PMC10788488

[33] Kang JY, Hur J, Kim S, et al. Clinical implications of discrepant results between genotypic MTBDRplus and phenotypic Lowenstein-Jensen method for isoniazid or rifampicin drug susceptibility tests in tuberculosis patients. J Thorac Dis. 2019;11(2):400–409. doi:10.21037/jtd.2019.01.58 30962983 PMC6409268

[34] Cui Z, Li Y, Cheng S, et al. Mutations in the embC-embA intergenic region contribute to Mycobacterium tuberculosis resistance to ethambutol. Antimicrob Agents Chemother. 2014;58(11):6837–6843. doi:10.1128/AAC.03285-14 25182646 PMC4249443

[35] Gomez-Gonzalez PJ, Campino S, Phelan JE, et al. Portable sequencing of Mycobacterium tuberculosis for clinical and epidemiological applications. Brief Bioinform. 2022;23(5):bbac256. 10.1093/bib/bbac256 PMC9487601 35894606

[36] Hall MB, Rabodoarivelo MS, Koch A, et al. Evaluation of Nanopore sequencing for Mycobacterium tuberculosis drug susceptibility testing and outbreak investigation: a genomic analysis. Lancet Microbe. 2023;4(2):e84–e92. doi:10.1016/S2666-5247(22)00301-9 36549315 PMC9892011

[37] Dheda K, Perumal T, Fox GJ. Asymptomatic tuberculosis: undetected and underestimated, but not unimportant. Lancet. 2025;405(10492):1797–1800. doi:10.1016/S0140-6736(25)00555-0 40127658

