# Supplementary material for: Rapid drug resistance prediction in positive Mycobacterium tuberculosis clinical samples using an extensive targeted next-generation sequencing panel
Source: Emerg Microbes Infect. 2026 Feb 3;15(1):2627072. doi: 10.1080/22221751.2026.2627072 (PMC12903939; doi:10.1080/22221751.2026.2627072)
Supplement: Supplementary_TB_TargetedSeq_Rosendal_revised.docx [file TEMI_A_2627072_SM2776.docx]

**Rapid drug resistance prediction in positive Mycobacterium tuberculosis clinical samples using an extensive targeted next-generation sequencing panel**

Ebba Rosendal^*1,2^, Joana Isidro^3^, Sofia Carneiro^4^, João Paulo Gomes^3,5^, Rita Macedo^*4^

^1^ ECDC Fellowship Programme, Public Health Microbiology path (EUPHEM), European Centre for Disease Prevention and Control (ECDC), Stockholm, Sweden

^2^ Infectious Diseases Department, National Institute of Health Doctor Ricardo Jorge, Lisbon, Portugal

^3^ Genomics and Bioinformatics Unit, Infectious Diseases Department, National Institute of Health Doctor Ricardo Jorge, Lisbon, Portugal

^4^ National Reference Laboratory for Mycobacteria, Infectious Diseases Department, National Institute of Health Doctor Ricardo Jorge, Lisbon, Portugal

^5^ Animal and Veterinary Research Center (CECAV), Faculty of Veterinary Medicine, Lusófona University - Lisbon University Centre, Lisbon, Portugal

* Corresponding authors

**Supplementary materials**

**Table S1.** Detailed information regarding amplicons and PCR pools, including genomic position, primer sequences and concentrations. Genomic position given in relation to *M. tuberculosis* H37Rv reference sequence (Genbank accession number: NC_000962.3).

| **PCR Pool** | **Gene** | **Amplicon**  **no.** | **Genomic position** | **Amplicon**  **length [nt]** | **Primer orientation** | **Primer sequence (5' to 3')** | **Primer concentration [µM]** | **Annealing temperature [°C]** |
| --- | --- | --- | --- | --- | --- | --- | --- | --- |
| 1 | *gyrA* | 1/1 | 7126 | 1085 | for | GGTTCGTGTGTTGCGTCAAGTG | 0.2 | 60 |
| 1 |  |  | 8210 | 1085 | rev | CTCAATGTTGGAAATGCCGGCC | 0.2 | 60 |
| 1 | *rpoB* | 1/2 | 760157 | 951 | for | AGACAAGGACATGACGTACGCG | 0.2 | 60 |
| 1 |  |  | 761107 | 951 | rev | ATGAATTGGCTCAGCTGGCTGG | 0.2 | 60 |
| 1 | *Rv0678* | 1/1 | 778717 | 1119 | for | GGAGCCGGAAACTTCGTACTCC | 0.2 | 60 |
| 1 |  |  | 779835 | 1119 | rev | CCACAACCGCTTCGATCCAGAT | 0.2 | 60 |
| 1 | *rrs* | 2/2 | 1472403 | 1123 | for | GGCGTAAAGAGCTCGTAGGTGG | 0.2 | 60 |
| 1 |  |  | 1473525 | 1123 | rev | GGAACAAGTCCGAGTGTTGCCT | 0.2 | 60 |
| 1 | *rrl* | 1/1 | 1475640 | 992 | for | GGTCGCAGAAACCAGTGAGGAG | 0.2 | 60 |
| 1 |  |  | 1476631 | 992 | rev | CTGACCGAACGTGGCTATCCAG | 0.2 | 60 |
| 1 | *katG* | 1/5 | 2152748 | 997 | for | GCATAGGTGCCGTCGCTATCAA | 0.2 | 60 |
| 1 |  |  | 2153744 | 997 | rev | ACGAGACGGGGATGAGGAGAAA | 0.2 | 60 |
| 1 | *pncA* | 1/2 | 2287649 | 1058 | for | CCTTGATATCGGGATAGCGCCG | 0.2 | 60 |
| 1 |  |  | 2288706 | 1058 | rev | CGAGTTGGTTTGCAGCTCCTGA | 0.2 | 60 |
| 1 | *ethA* | 2/2 | 4326523 | 1130 | for | CTCGACCTTCCCGTGACGAATG | 0.2 | 60 |
| 1 |  |  | 4327652 | 1130 | rev | GGACGGTCCTCGAGAAGGTTCT | 0.2 | 60 |
| 1 | *alr* | 1/2 | 3839881 | 1136 | for | GTTGTGGTCCAGCAGTCGGTAG | 0.2 | 60 |
| 1 |  |  | 3841016 | 1136 | rev | CAACTCGACGAACTGTTGCACG | 0.2 | 60 |
| 1 | *ald* | 2/2 | 3087079 | 1034 | for | CGACACGGGCAGATCTTGTTCA | 0.2 | 60 |
| 1 |  |  | 3088112 | 1034 | rev | GAGGTCCACCAGATGACGAAGC | 0.2 | 60 |
| 1 | *inhA* | 1/2 | 1673288 | 923 | for | GGGATCCGTCATGGTCGAAGTG | 0.2 | 60 |
| 1 |  |  | 1674210 | 923 | rev | TCCTGTCATGTGCGTCCTTGTG | 0.2 | 60 |
| 1 | *folC* | 1/1 | 2746865 | 1089 | for | CAGCAGCACCTCCATGACCTTC | 0.2 | 60 |
| 1 |  |  | 2747953 | 1089 | rev | CTCGGATCTGAGCAACCAGGTG | 0.2 | 60 |
| 2 | *gyrB* | 1/1 | 6362 | 1033 | for | TTGGGCAACACCGAGGTCAAAT | 0.4 | 63 |
| 2 |  |  | 7394 | 1033 | rev | ATAGTCGATGTAGCTGCGCTGC | 0.4 | 63 |
| 2 | *tlyA* | 1/1 | 1917769 | 1145 | for | CTGACCAGATCCGTGCCGAAAT | 0.2 | 63 |
| 2 |  |  | 1918913 | 1145 | rev | CATGTCGTCGGGAGCCAGATG | 0.2 | 63 |
| 2 | *katG* | 3/5 | 2153917 | 1008 | for | GTTCATCACCTTGTCCCAGGCA | 0.4 | 63 |
| 2 |  |  | 2154924 | 1008 | rev | CTGGAACACCCCGAGGAATTGG | 0.4 | 63 |
| 2 | *katG* | 5/5 | 2155198 | 1055 | for | GGTGCCATACGAGCTCTTCCAG | 0.2 | 63 |
| 2 |  |  | 2156252 | 1055 | rev | CTCGGACCATAACGGCTTCCTG | 0.2 | 63 |
| 2 | *eis* | 1/1 | 2714551 | 1007 | for | CATGGAGTCGAGGCCAATCAGG | 0.2 | 63 |
| 2 |  |  | 2715557 | 1007 | rev | CATGGGACCGGTACTTGCTCTG | 0.2 | 63 |
| 2 | *ethA* | 1/2 | 4325796 | 1134 | for | GCATCGACATCGGCATCAAAGC | 0.2 | 63 |
| 2 |  |  | 4326929 | 1134 | rev | AGAACATCGTCGTGATCGGCAG | 0.2 | 63 |
| 2 | *pncA* | 2/2 | 2288375 | 1142 | for | TAGCGTAGGAAGGCGGGAATGA | 0.4 | 63 |
| 2 |  |  | 2289516 | 1142 | rev | GTGATATGGGGGATCGGCATCG | 0.4 | 63 |
| 2 | *ald* | 1/2 | 3086641 | 953 | for | GGATTCTGCGGTCCACCTCATC | 0.2 | 63 |
| 2 |  |  | 3087593 | 953 | rev | TGCGCGACAAGTGAATTCGAGA | 0.2 | 63 |
| 3 | *rpoB* | 2/2 | 760572 | 1011 | for | CTGGAGAAGGACAACACCGTCG | 0.2 | 60 |
| 3 |  |  | 761582 | 1011 | rev | CTCCAGGAAGGGAATCATCGCG | 0.2 | 60 |
| 3 | *rplC* | 1/1 | 800798 | 1094 | for | TGGACAGAGCAATGGCACGAAA | 0.2 | 60 |
| 3 |  |  | 801891 | 1094 | rev | TCGACGGGTTTTGACCTTCCAC | 0.2 | 60 |
| 3 | *katG* | 2/5 | 2153180 | 1117 | for | GCTGTCACTGCATTGCTGTCAC | 0.4 | 60 |
| 3 |  |  | 2154296 | 1117 | rev | AGTACATGCTGCTCGACAAGGC | 0.4 | 60 |
| 3 | *katG* | 4/5 | 2154550 | 1092 | for | TTCCAGGGTGCGAATGACCTTG | 0.2 | 60 |
| 3 |  |  | 2155641 | 1092 | rev | CTCTCATGGGCGGACCTGATTG | 0.2 | 60 |
| 3 | *ddn* | 1/1 | 3986435 | 1024 | for | GGAGAAGGCCGGCATGAAGATC | 0.2 | 60 |
| 3 |  |  | 3987548 | 1024 | rev | ACAAGGGCGTGAAATGGGATCC | 0.2 | 60 |
| 3 | *thyA* | 1/1 | 3073507 | 1136 | for | ATGGTGATCTCCCGGAAATGCG | 0.2 | 60 |
| 3 |  |  | 3074642 | 1136 | rev | CATGGCCTCCGTTGTACTCCTG | 0.2 | 60 |
| 3 | *alr* | 2/2 | 3840560 | 1061 | for | CACGAATCGATTTCACCAGCGC | 0.6 | 60 |
| 3 |  |  | 3841620 | 1061 | rev | GTCAAGCTACGCTCCCGTCAAT | 0.6 | 60 |
| 3 | *inhA* | 2/2 | 1673601 | 1122 | for | GCTGTTTGGCGTCGAATGTGAC | 0.2 | 60 |
| 3 |  |  | 1674722 | 1122 | rev | AACCTGTTGACCGACTCCAACG | 0.2 | 60 |
| 3 | *rrs* | 1/2 | 1471673 | 1019 | for | CGGAAACAAGCAAGCGTGTTGT | 0.2 | 60 |
| 3 |  |  | 1472691 | 1019 | rev | CGGATCCCAAGGAAGGAAACCC | 0.2 | 60 |
| 3 | *embB* | 1/1 | 4247330 | 805 | for | ACCGACGCCGTGGTGATATTCG | 0.1 | 60 |
| 3 |  |  | 4248134 | 805 | rev | CGGTGATCAAAAAGCCGAAGCG | 0.1 | 60 |

**Table S3**. Genotypic DST from tNGS compared to phenotypic results for all samples using <30x depth of coverage in at least one POI for that antibiotic as cut-off for undetermined result. Categorical agreement (CA) was calculated wither with (total) or without undetermined results. Sensitivity and specificity calculated excluding undetermined results. The 95% confidential interval (95 CI) for sensitivity and specificity is displayed in brackets.

|  | **pDST resistant** | | | |  | **pDST susceptible** | | | |  |  |  |  |
| --- | --- | --- | --- | --- | --- | --- | --- | --- | --- | --- | --- | --- | --- |
|  |  | **tNGS results** | | |  |  | **tNGS results** | | | **CA total**  **(%)** | **CA**  **(%)** | **Sensitivity**  **[95 CI]** | **Specificity**  **[95 CI]** |
| **Antibiotic** | **Total** | **R** | **S** | **U** |  | **Total** | **R** | **S** | **U** |  |  |  |  |
| INH | 20 | 14 | 2 | 4 |  | 51 | 1 | 33 | 17 | 66.2 | 94.0 | 0.88 [0.62, 0.98] | 0.97 [0.85, 1] |
| PZA | 2 | 2 | 0 | 0 |  | 69 | 0 | 65 | 4 | 94.4 | 100 | 1.0 [0.16, 1] | 1.0 [0.94, 1] |
| EMB | 3 | 2 | 1 | 0 |  | 32 | 0 | 32 | 0 | 97.1 | 97.1 | 0.67 [0.09, 0.99] | 1.0 [0.89, 1] |
| RIF | 4 | 4 | 0 | 0 |  | 67 | 0 | 52 | 15 | 78.9 | 100 | 1.0 [0.40, 1] | 1.0 [0.93, 1] |
| **Total** | **29** | **22** | **3** | **4** |  | **219** | **1** | **182** | **36** | **82.3** | **98.1** | **0.88 [0.69, 0.97]** | **0.99 [0.97, 1]** |

**Table S4.** Genotypic DST results for first-line antibiotics using targeted next-generation sequencing (tNGS) compared to phenotypic DST results (pDST). Categorical agreement (CA) calculated wither with (total) or without undetermined results; defined as below 10x depth of coverage in at least one POI for that antibiotic.

|  | **pDST resistant** | | | |  | **pDST susceptible** | | | |  |  |
| --- | --- | --- | --- | --- | --- | --- | --- | --- | --- | --- | --- |
|  |  | **tNGS results** | | |  |  | **tNGS results** | | | **CA total**  **(%)** | **CA**  **(%)** |
| **Antibiotic** | **Total** | **R** | **S** | **U** |  | **Total** | **R** | **S** | **U** |  |  |
| MFX | 0 | 0 | 0 | 0 |  | 4 | 1 | 3 | 0 | 75% | 75% |
| LFX | 0 | 0 | 0 | 0 |  | 4 | 1 | 3 | 0 | 75% | 75% |
| BDQ | 0 | 0 | 0 | 0 |  | 4 | 0 | 4 | 0 | 100% | 100% |
| DLM | 0 | 0 | 0 | 0 |  | 4 | 0 | 4 | 0 | 100% | 100% |
| LZD | 0 | 0 | 0 | 0 |  | 4 | 0 | 4 | 0 | 100% | 100% |
| AMK | 0 | 0 | 0 | 0 |  | 4 | 1 | 3 | 0 | 75% | 75% |
| KAN | 1 | 1 | 0 | 0 |  | 3 | 0 | 3 | 0 | 100% | 100% |
| CAP | 0 | 0 | 0 | 0 |  | 4 | 1 | 3 | 0 | 75% | 75% |
| CFZ | 0 | 0 | 0 | 0 |  | 4 | 0 | 4 | 0 | 100% | 100% |
| ETH | 2 | 2 | 0 | 0 |  | 2 | 0 | 2 | 0 | 100% | 100% |
| PAS | 1 | 0 | 0 | 1 |  | 3 | 0 | 3 | 0 | 75% | 100% |
| CYCLO | 1 | 0 | 1 | 0 |  | 3 | 0 | 3 | 0 | 75% | 75% |
| **Total** | **5** | **3** | **1** | **1** |  | **43** | **4** | **39** | **0** | **89%** | **93%** |

**Table S5**. Genotypic DST from tNGS compared to genotypic DST from whole genome sequencing (WGS) for all samples with WGS results (n=53) using 30x depth of coverage in at least one POI for that antibiotic as cut-off for undetermined result. Categorical agreement (CA) was calculated wither with (total) or without undetermined results. Sensitivity and specificity calculated excluding undetermined results. The 95% confidential interval (95 CI) for sensitivity and specificity is displayed in brackets.

|  | **WGS resistant** | | | |  | **WGS susceptible** | | | |  |  |  |  |
| --- | --- | --- | --- | --- | --- | --- | --- | --- | --- | --- | --- | --- | --- |
|  |  | **tNGS results** | | |  |  | **tNGS results** | | | **CA total**  **(%)** | **CA**  **(%)** | **Sensitivity**  **[95 CI]** | **Specificity**  **[95 CI]** |
| **Antibiotic** | **Total** | **R** | **S** | **U** |  | **Total** | **R** | **S** | **U** |  |  |  |  |
| INH | 12 | 10 | 0 | 2 |  | 41 | 0 | 26 | 15 | 67.9 | 100 | 1.0 [0.69, 1] | 1.0 [0.87, 1] |
| PZA | 2 | 2 | 0 | 0 |  | 51 | 0 | 49 | 2 | 96.2 | 100 | 1.0 [0.16, 1] | 1.0 [0.93, 1] |
| EMB | 3 | 2 | 1 | 0 |  | 24 | 0 | 24 | 0 | 96.3 | 96.3 | 0.67 [0.09, 0.99] | 1.0 [0.86, 1] |
| RIF | 4 | 4 | 0 | 0 |  | 49 | 0 | 37 | 12 | 77.4 | 100 | 1.0 [0.40, 1] | 1.0 [0.91, 1] |
| MFX | 1 | 1 | 0 | 0 |  | 52 | 0 | 45 | 7 | 86.8 | 100 | 1.0 [0.03, 1] | 1.0 [0.92, 1] |
| LFX | 1 | 1 | 0 | 0 |  | 52 | 0 | 45 | 7 | 86.8 | 100 | 1.0 [0.03, 1] | 1.0 [0.92, 1] |
| BDQ | 0 | 0 | 0 | 0 |  | 53 | 0 | 51 | 2 | 96.2 | 100 | NA | 1.0 [0.93, 1] |
| DLM | 0 | 0 | 0 | 0 |  | 53 | 0 | 44 | 9 | 83 | 100 | NA | 1.0 [0.92, 1] |
| LZD | 0 | 0 | 0 | 0 |  | 53 | 0 | 43 | 10 | 81.1 | 100 | NA | 1.0 [0.92, 1] |
| AMK | 0 | 0 | 0 | 0 |  | 53 | 1 | 46 | 6 | 86.8 | 97.9 | NA | 0.98 [0.89, 1] |
| KAN | 0 | 0 | 0 | 0 |  | 53 | 1 | 46 | 6 | 86.8 | 97.9 | NA | 0.98 [0.89, 1] |
| CAP | 1 | 0 | 0 | 1 |  | 52 | 1 | 44 | 7 | 83 | 97.8 | NA | 0.98 [0.88, 1] |
| CFZ | 0 | 0 | 0 | 0 |  | 53 | 0 | 51 | 2 | 96.2 | 100 | NA | 1.0 [0.93, 1] |
| ETH | 4 | 4 | 0 | 0 |  | 49 | 0 | 34 | 15 | 71.7 | 100 | 1.0 [0.40, 1] | 1.0 [0.9, 1] |
| PAS | 1 | 0 | 0 | 1 |  | 52 | 0 | 42 | 10 | 79.2 | 100 | NA | 1.0 [0.92, 1] |
| CYCLO | 0 | 0 | 0 | 0 |  | 53 | 0 | 40 | 13 | 75.5 | 100 | NA | 1.0 [0.91, 1] |
| **Total** | **29** | **24** | **1** | **4** |  | **793** | **3** | **667** | **123** | **84.1** | **99.4** | **0.96 [0.80, 1]** | **1.0 [0.99, 1]** |

**Table S6**. Non-resistance associated mutations determined by WGS and the corresponding results by tNGS. Where results are different the results using >10x and 30x depth of coverage as cut-off for undetermined result both are included as 10x-result/30x-result.

|  |  |  | **Present in WGS** | | | |  |  |
| --- | --- | --- | --- | --- | --- | --- | --- | --- |
|  |  |  |  | **tNGS results**  **(10x/30x threshold)** | | |  |  |
| **Gene** | **Mutation/**  **substitution** | **Genome position** | **Total** | **P** | **A** | **U** |  | **Undetermined**  **results**  **(10x/30x threshold)** |
| *gyrB* | p.Pro400Arg | 6438 | 1 | 1 | 0 | 0 |  | 0% |
| *gyrB* | p.Val457Leu | 6608 | 1 | 1 | 0 | 0 |  | 0% |
| *gyrB* | c.1578G>A | 6817 | 3 | 2 | 1 | 0 |  | 0% |
| *gyrB* | p.Glu592Gly | 7014 | 1 | 1 | 0 | 0 |  | 0% |
| *gyrA* | p.Glu21Gln | 7362 | 50 | 49/46 | 0 | 1/4 |  | 2%/8% |
| *gyrA* | p.Ser95Thr | 7585 | 45 | 41/40 | 2/1 | 2/1 |  | 4%/9% |
| *gyrA* | p.Arg222Trp | 7965 | 3 | 2 | 0 | 1 |  | 33% |
| *gyrA* | p.Gly247Ser | 8040 | 3 | 3 | 0 | 0 |  | 0% |
| *mmpR5* | p.Asp5Gly | 779003 | 1 | 1 | 0 | 0 |  | 0% |
| *rplC* | p.Ala31Thr | 800899 | 1 | 1 | 0 | 0 |  | 0% |
| *rplC* | p.Ala103Thr | 801115 | 1 | 1 | 0 | 0 |  | 0% |
| *rplC* | c.546G>A | 801354 | 1 | 0 | 0 | 1 |  | 100% |
| *rrs* | n.-121C>T | 1471725 | 1 | 0 | 0 | 1 |  | 100% |
| *rrs* | n.-13G>A | 1471833 | 1 | 0 | 0 | 1 |  | 100% |
| *rrs* | n.-4T>G | 1471842 | 1 | 1 | 0 | 0 |  | 0% |
| *rrs* | n.1286G>C | 1473131 | 1 | 0 | 1 | 0 |  | 0% |
| *rrl* | n.2352G>A | 1476009 | 1 | 0 | 1 | 0 |  | 0% |
| *inhA* | c.9A>C | 1674210 | 1 | 1 | 0 | 0 |  | 0% |
| *tlyA* | c.33A>G | 1917972 | 50 | 49/46 | 0 | 1/4 |  | 2%/8% |
| *tlyA* | p.Asp48Asn | 1918081 | 1 | 1 | 0 | 0 |  | 0% |
| *katG* | c.1926G>A | 2154186 | 1 | 0 | 1 | 0 |  | 0% |
| *katG* | p.Arg463Leu | 2154724 | 8 | 7 | 1 | 0 |  | 0% |
| *pncA* | p.Ala79Val | 2289006 | 1 | 1 | 0 | 0 |  | 0% |
| *pncA* | c.225T>C | 2289017 | 1 | 1 | 0 | 0 |  | 0% |
| *pncA* | c.195C>T | 2289047 | 1 | 1 | 0 | 0 |  | 0% |
| *pncA* | c.-125delC | 2289365 | 2 | 1 | 1 | 0 |  | 0% |
| *folC* | p.Val193Ile | 2747022 | 1 | 0 | 1 | 0 |  | 0% |
| *folC* | c.555C>T | 2747044 | 1 | 0 | 1 | 0 |  | 0% |
| *folC* | c.-194G>T | 2747792 | 1 | 1 | 0 | 0 |  | 0% |
| *thyA* | c.726C>T | 3073746 | 1 | 1 | 0 | 0 |  | 0% |
| *thyA* | p.Thr202Ala | 3073868 | 19 | 16 | 2/1 | 1/2 |  | 5%/11% |
| *thyA* | c.438T>C | 3074034 | 1 | 1 | 0 | 0 |  | 0% |
| *ald* | c.-32T>C | 3086788 | 45 | 38/33 | 2 | 5/10 |  | 11%/22% |
| *ald* | p.Gln56His | 3086987 | 1 | 1 | 0 | 0 |  | 0% |
| *alr* | c.702A>G | 3840719 | 2 | 2 | 0 | 0 |  | 0% |
| *alr* | c.-13G>C | 3841433 | 1 | 1 | 0 | 0 |  | 0% |
| *embB* | p.Glu378Ala | 4247646 | 1 | 1/0 | 0 | 0/1 |  | 0%/100% |
| *embB* | c.1479G>A | 4247992 | 1 | 0 | 0 | 1 |  | 100% |
| *ethA* | p.Asn345Lys | 4326439 | 1 | 1 | 0 | 0 |  | 0% |
| *ethR* | c.-363C>T | 4327186 | 1 | 1 | 0 | 0 |  | 0% |
| *ethA* | c.24C>T | 4327450 | 1 | 1 | 0 | 0 |  | 0% |
| **Total** | **-** | **-** | **260** | **231/218** | **14/12** | **15** |  | **6%/12%** |
